# Nanoscale Characterization of Photocurrent and Photovoltage in Polycrystalline Solar Cells

**DOI:** 10.1021/acs.jpcc.3c00239

**Published:** 2023-06-07

**Authors:** Dongheon Ha, Yohan Yoon, Ik Jae Park, Luis Torres Cantu, Aries Martinez, Nikolai Zhitenev

**Affiliations:** † Department of Physics, Eastern Illinois University, Charleston, Illinois 61920, United States; ‡ Physical Measurement Laboratory, National Institute of Standards and Technology, Gaithersburg, Maryland 20899, United States; § Institute for Research in Electronics and Applied Physics, University of Maryland, College Park, Maryland 20742, United States; ∥ Department of Materials Science and Engineering, Korea Aerospace University, Goyang-si, Gyeonggi-do 10540, Korea; ⊥ Department of Materials Physics, Sookmyung Women’s University, Seoul 04310, Korea

## Abstract

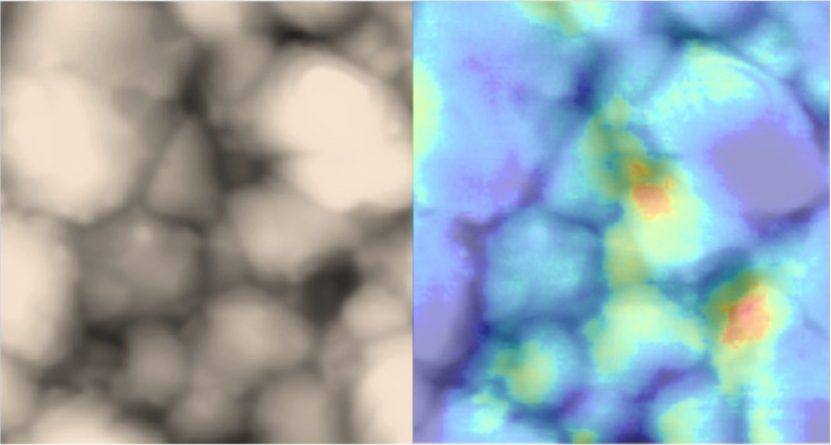

We investigate the role of grain structures in nanoscale carrier dynamics of polycrystalline solar cells. By using Kelvin probe force microscopy (KPFM) and near-field scanning photocurrent microscopy (NSPM) techniques, we characterize nanoscopic photovoltage and photocurrent patterns of inorganic CdTe and organic–inorganic hybrid perovskite solar cells. For CdTe solar cells, we analyze the nanoscale electric power patterns that are created by correlating nanoscale photovoltage and photocurrent maps on the same location. Distinct relations between the sample preparation conditions and the nanoscale photovoltaic properties of microscopic CdTe grain structures are observed. The same techniques are applied for characterization of a perovskite solar cell. It is found that a moderate amount of PbI_2_ near grain boundaries leads to the enhanced photogenerated carrier collections at grain boundaries. Finally, the capabilities and the limitations of the nanoscale techniques are discussed.

## Introduction

As traditional energy sources are continuously used up and/or polluting the environment, it is imperative to develop next-generation renewable energy sources, including solar cells. While it will take time for solar cells to fully replace or significantly supplement the conventional energy options, relentless efforts are being made to improve cost efficiency of solar cells. Considerable recent progress driven by material development^1−12^ and nanophotonic engineering^13−27^ raised expectations for a significant amount of electricity generated by solar cells in the near future. As a promising example, the cells based on new emerging materials such as perovskite solar cells are rapidly improving with the best cell efficiency greater than ≈26%,^28^ exceeding the performance of traditional solar cell technologies. A significant enhancement of optical and electrical properties of solar cells has also been achieved with nanophotonic materials, such as metallic particles,^15,16,21,22^ dielectric scatterers,^13,17,23,27^ and semiconductor nanostructures.^14,18−20,24−26^

As solar technologies are gradually improving, it is essential to develop new characterization techniques to advance our understanding of newly developed cells. In-depth understanding of cell operation acts as a foundation for further improving photovoltaic technologies. To thoroughly analyze the photovoltaic characteristics, we need to investigate the fundamental interaction between photons, electrons, and materials at the nanoscale because photogenerated carrier generations and collections take place on this scale. This brings forward the need of developing nanoscale characterization techniques.

**Figure 1:**
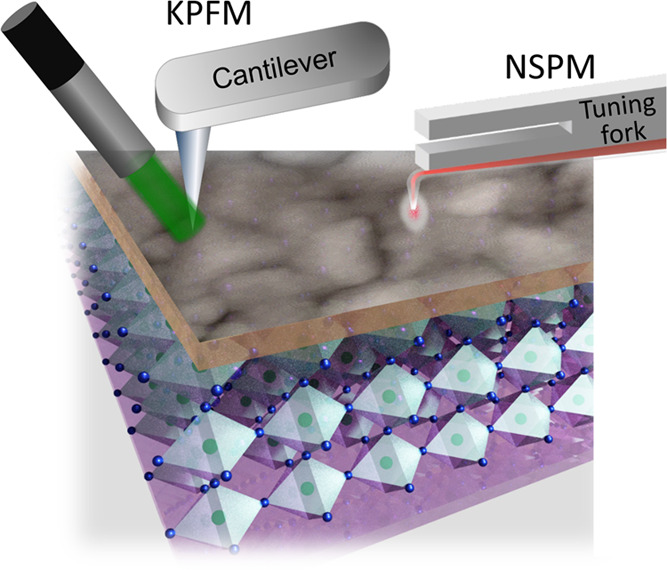
Schematic illustrating KPFM and NSPM measurements on a polycrystalline solar cell, such as a perovskite solar cell. In KPFM measurements, a conductive AFM cantilever detects surface contact potential, and light is illuminated onto a relatively large area of a sample. In NSPM, photogenerated carriers are collected via macroscale sample electrodes, while a laser light is injected through a small tapered optical fiber attached below a tuning fork-based AFM probe.

Several nanoscale characterization methods have been actively applied to solar cell materials and devices to reveal nanoscale photoresponses. Among them, the methods that can directly monitor photocurrent and photovoltage at the nanoscale stand out, as these two optoelectronic properties are the most critical factors that affect the efficiency of solar cells. A few atomic force microscopy (AFM)-based methods have been developed for nanoscale photocurrent measurements.^29−41^ The techniques differ in the light injection and carrier collection mechanisms. For example, photoconductive AFM injects light over a relatively large sample surface area and collects photogenerated carriers into a conductive AFM cantilever.^29−33,35−37,40^ Here, we use near-field scanning photocurrent microscopy (NSPM) that utilizes a small tapered optical fiber as a local light source and collects photogenerated carriers through macroscale sample electrodes (see Figure 1).^34,38,39,41^

To obtain nanoscale photovoltage information of solar cells, various techniques have been used.^35,42−46^ In this work, we use Kelvin probe force microscopy (KPFM), one of the most sensitive scanning probe techniques, to measure the photoinduced surface contact potential of solar cell devices having nanoscale spatial resolution. In KPFM measurements, an AFM cantilever detects the contact potential difference (*V*_CPD_) of a sample by detecting the potential offset between a probe tip (i.e., a conductive AFM cantilever) and sample surface (see Figure 1). KPFM is performed in a two-pass imaging mode where a conductive cantilever images the surface morphology in tapping mode in the first pass and then lifts above the surface by a constant height to quantitatively measure the sample’s surface potential in the second pass.

In this study, we apply these two nanoscale characterization methods, KPFM and NSPM, to investigate the nanoscale photoresponses of polycrystalline thin-film solar cells. By using both techniques, we obtained nanoscale photovoltage and photocurrent maps of the cells. The selected AFM-based measurement techniques are versatile, so we applied them to various polycrystalline photovoltaic cells, including inorganic cadmium telluride (CdTe) solar cells and organic–inorganic hybrid perovskite solar cells. We found that the spatial patterns of both quantities depend sensitively on the processing/fabrication history with the contrast between the grain cores and grain boundaries being reversed upon treatment and/or opposite for photocurrent and photovoltage. As a result, a single type of measurement appears to be an insufficient predictor of the cell efficiency and/or usefulness of a particular processing condition. Thus, we also determined nanoscopic local electric power patterns of CdTe solar cells by combining nanoscale photovoltage and photocurrent images on the same location. Finally, we discuss the capabilities and the limitations of the nanoscale measurement techniques used in this study.

## Methods

### Near-Field Scanning Photocurrent Microscopy (NSPM)

Customized tuning fork-based atomic force microscopy (AFM) probes were used in this study. A tapered optical fiber having a small aperture (a diameter of around 150–250 nm) was used to locally inject light. The optical fiber was coupled with a diode laser (a wavelength of ≈520 nm). The measured electrical signal was amplified by a preamplifier and was low-pass-filtered using a lock-in amplifier to increase the signal-to-noise ratio. While in noncontact mode raster scanning, both topography and photocurrent images were acquired from the same location of the sample.

### Kelvin Probe Force Microscopy (KPFM)

In tapping mode AFM, a platinum iridium (Pt/Ir)-coated cantilever (force constant: 3 N/m, resonance frequency: 75 kHz) was used to simultaneously acquire both topography and surface potential images. The cantilever was connected to the rear contact of a sample to eliminate any charging effect of the sample. To illuminate the sample, we used a bench top laser. A FC/PC fiber was connected to the output port of the laser, and the other end was connected with a collimator for uniform light illumination.

### CdTe Cell Fabrication

CdTe thin-film cells composed of CdS and CdTe layers were deposited on a fluorine-doped tin oxide (FTO)-coated float glass substrate. Prior to the deposition process, the substrates were heated up to two different temperatures of 310 and 500 °C, referred to low-temperature (LT) and high-temperature (HT) in this work, respectively. The average thickness of the CdTe and CdS layers are 4–5 μm and ≈150 nm, respectively. After the CdTe deposition, Cl activation treatment was carried out using a saturated methanolic CdCl_2_ solution at 400° for 25 min. A gold layer was sputtered using a DC magnetron sputtering process to form a contact having a thickness of 130 nm.

### Perovskite Cell Fabrication

An indium tin oxide (ITO) layer on a glass substrate was rinsed by sonification in water, acetone, and ethyl alcohol in sequence. A solution containing a 1 mmol of nickel(II) acetate tetrahydrate in ethyl alcohol was spin-coated on the substrate at 4000 rpm for 45 s, followed by annealing at 300 °C for 1 h. The PbI_2_ and methylammonium iodide with a 1:1 molar ratio was dissolved in a mixture of *N*,*N*-dimethylformamide and dimethyl sulfoxide (DMSO) (9:1 by volume) and was spin-coated at 4000 rpm for 25 s. Antisolvent (diethyl ether) was quickly dropped on the substrate during the spinning process to form PbI_2_-MAI-DMSO intermediate phases. For crystallization of perovskite, the substrate was annealed at 100 °C for 10 min. The electron transport layer composed of a 50 nm thick zinc oxide nanoparticle (ZnO NP) layer and a 50 nm thick phenyl-C_61_-butyric acid methyl ester ([60]PCBM) layer was added. The PCBM layer was formed by spinning of the [6,6]-phenyl-C_61_-butyric acid methyl ester solution (20 mg/mL in chlorobenzene) on top of the perovskite layer. Finally, a 120 nm thick Ag electrode was deposited by thermal evaporation.

## Results and Discussion

We examined three different types of CdTe cells. Two different CdTe cells with p-type CdTe and n-type cadmium sulfide (CdS) were fabricated specifically for this project. During the fabrication, the cell substrates were annealed at different temperatures. A low-temperature (LT) sample and a high-temperature (HT) sample were heated at 310 and 500 °C, respectively, during the deposition of CdTe layer. Chlorine (Cl) activation treatment was subsequently carried out to reduce the recombination centers at grain boundaries.^41^ The motivation for investigating CdTe solar cells with different substrate temperatures is to lower the energy budget of the fabrication process. The third cell is an optimized, commercially available CdTe solar cell, and the Cl activation treatment was also applied to this sample. This set of samples was used to study the role of microscopic CdTe grain structures and to find the correlation between macroscale photovoltaic properties and nanoscale photoresponses.

**Figure 2:**
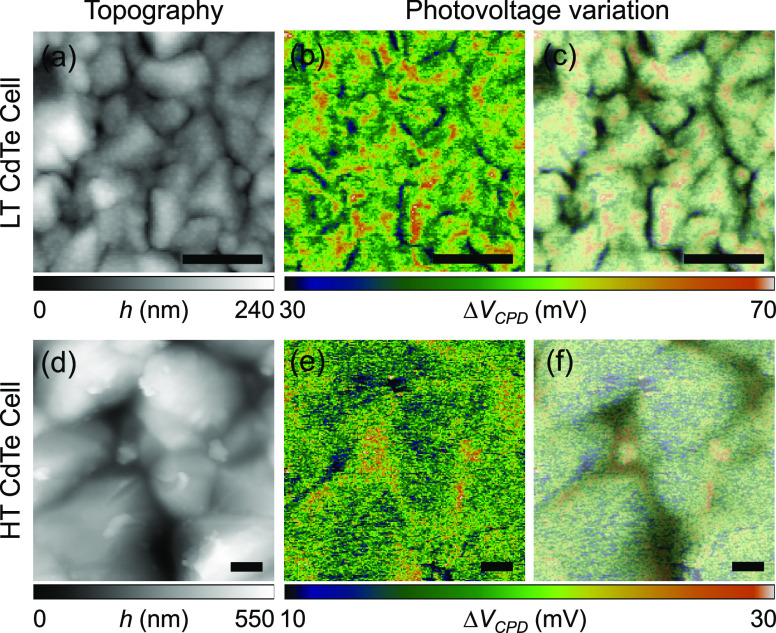
AFM topography images of (a) LT CdTe and (d) HT CdTe samples. Measured photovoltage information for (b) LT CdTe and (e) HT CdTe samples. AFM topography images and photovoltage images are overlaid to more effectively illustrate the correlation between grain structures and photoresponse. AFM topography-overlaid photovoltage images for (c) LT CdTe and (f) HT CdTe samples. Scale bar: 1 μm.

**Figure 3:**
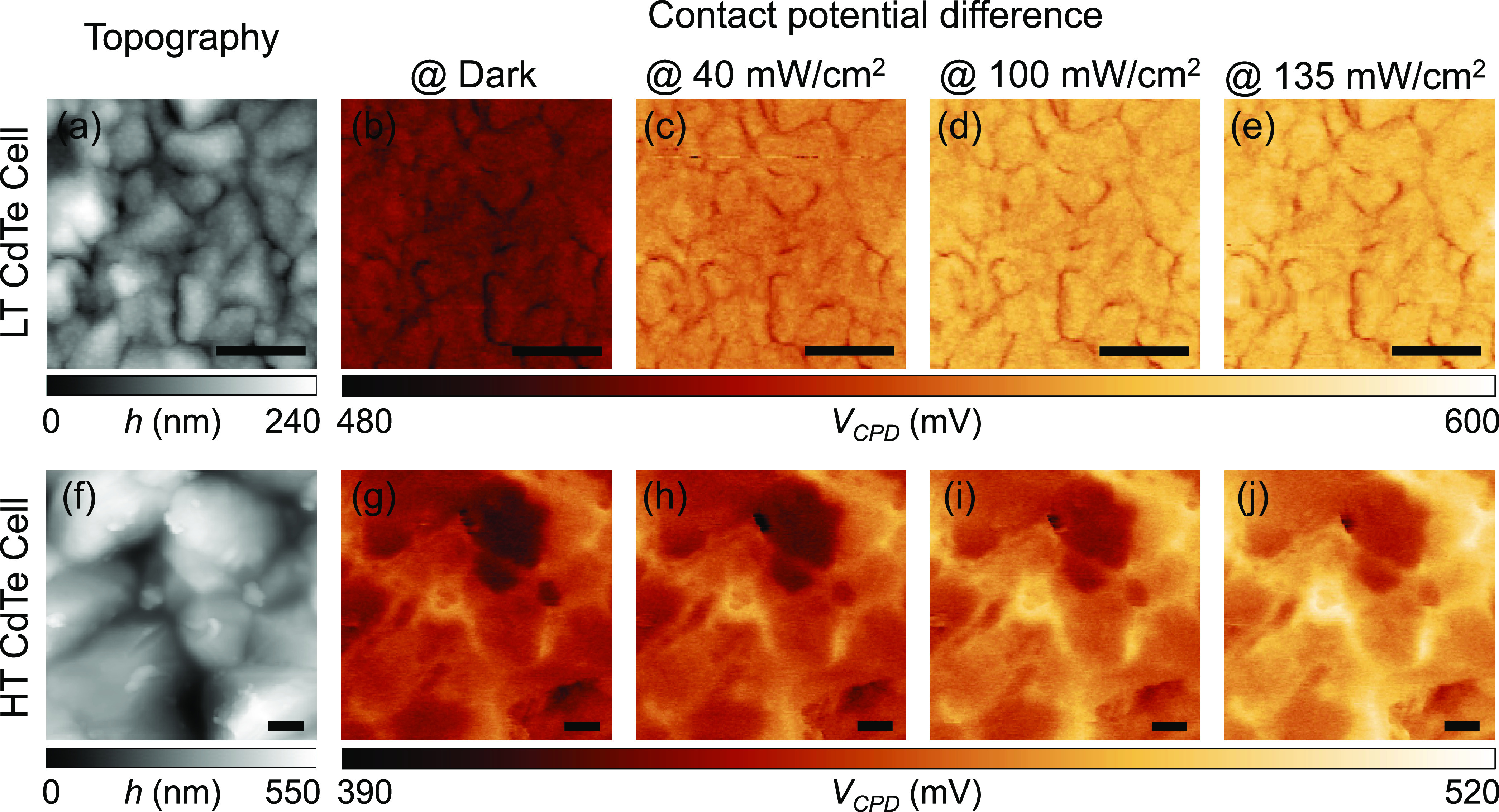
Topography of (a) LT CdTe and (f) HT CdTe samples. Contact potential images of the LT CdTe cell (b) at dark and (c–e) under light illumination. Contact potential images of the HT CdTe cell (g) at dark and (h–j) under light illumination. Three different illumination conditions were applied: (c, h) 40 mW/cm^2^, (d, i) 100 mW/cm^2^, and (e, j) 135 mW/cm^2^. Scale bar: 1 μm.

Macroscale photovoltaic properties of the three CdTe samples are summarized in Table 1. We first compare LT and HT samples. As the sample annealing temperature is increased from 310 to 500 °C, the short-circuit current density is decreased from 20.8 to 20.1 mA/cm^2^, while the open-circuit voltage is increased from 760 to 800 mV. We attribute these changes to the enlarged average grain size of the HT sample.^41^ While forming larger grains, the density of microscopic carrier recombination sites (i.e., grain boundaries) is reduced, and therefore, the open-circuit voltage of the entire sample increases.^47,48^ It is found that the average grain size of the LT sample is less than ≈1 μm and the HT sample is greater than ≈3 μm (see topography images in Figures 2 and 3). Along with the improved fill factor (from 58 to 69%), the increased open-circuit voltage leads to the increased power conversion efficiency (from 9.1 to 11.1%). However, the optimized CdTe cell in which a medium annealing temperature, 370 °C, was used shows both a higher short-circuit current density and an increased open-circuit voltage, ultimately leading to a better power conversion efficiency than the efficiencies of first two CdTe samples.^49^

**Table 1:** Experimentally Determined Macroscale Photovoltaic Properties of CdTe Samples^a^

	short-circuit current density (mA/cm^2^)	open-circuit voltage (mV)	fill factor (%)	cell efficiency (%)
LT CdTe sample	20.8 ± 0.22	760 ± 0.9	58 ± 0.9	9.1 ± 0.09
HT CdTe sample	20.1 ± 0.15	800 ± 1.1	69 ± 1.3	11.1 ± 0.07
optimized CdTe sample	23.3 ± 0.10	820 ± 1.0	64 ± 1.0	12.0 ± 0.10

a The uncertainty in this table is one standard deviation of measurements of at least six identical samples with the same fabrication condition.

It is worth mentioning that the thickness of the LT and HT CdTe cells is on the order of 4–5 μm, which is thicker than that of the optimized CdTe sample. In the optimized CdTe sample, the thicknesses of p-type CdTe layers and n-type CdS layers are ≈3 μm and ≈50 nm, respectively. Therefore, in the optimized cell, the minority carrier diffusion length is comparable to the CdTe grain size and film thickness,^50−53^ meaning that most of photogenerated minority carriers can be efficiently collected via both top and bottom electrodes before recombining. Note that the average grain size of the optimized CdTe solar cell is on the order of 1.5–3 μm, larger than that of the LT sample and smaller than that of the HT sample. The increased film thickness of the LT and HT samples is detrimental to minority carrier collection, as the film thickness exceeds the minority carrier diffusion length of CdTe solar cells.^53^ The thickness of the sample is also related to the effect of Cl activation treatment, affecting defect passivation at both grain boundaries and intragrains. Overall, the optimized sample has a thickness that advances carrier transport and collection. For such reason, the short-circuit current densities of the LT and HT samples are decreased compared with that of the optimized sample, as shown in Table 1.

When we compare the HT sample to the optimized one, the HT sample exhibits the lower power conversion efficiency despite the fact that the fill factor of the sample is higher. The higher fill factor in the HT CdTe cell can be attributed to its larger grain size compared to the optimized sample, originating from the elevated annealing temperature. The larger grain effectively decreases the density of grain boundaries, and thus, the shunt loss of the device is suppressed. However, the HT CdTe cell has a lower short-circuit current density compared to other samples due to Cl segregation at grain boundaries originated from the higher annealing temperature [seeFigure S1 in the Supporting Information for energy-dispersive X-ray spectroscopy in scanning transmission electron microscopy (EDX/STEM) results]. The Cl segregation at grain boundaries in the HT CdTe sample hinders effective carrier collections at grain boundaries, reducing the short-circuit density of the device. The trade-off between the shunt loss and the short-circuit density explains why the optimized sample achieved the best cell efficiency even though its fill factor was not the highest among all samples.

To understand how the grain structures and sizes derived from different fabrication conditions affect micro/nanoscopic photogenerated carrier dynamics, we examine nanoscale local photovoltage maps obtained by KPFM measurements. For KPFM measurements, we applied platinum/iridium (Pt/Ir)-coated conductive AFM probes. The wavelength of the injected light is ≈520 nm. Figure 2 shows the topography and photovoltage (Δ*V*_CPD_) images of the LT and HT CdTe samples. The photovoltage (Δ*V*_CPD_) images in Figure 2b,e were obtained by subtracting dark surface contact potential difference (*V*_CPD_ at dark in Figure 3b,g) from photoexcited surface contact potential difference (*V*_CPD_ in Figure 3d,i) of the samples under light illumination, respectively.

The potential dependence on the light intensity is shown in Figure 3. The contact potential difference image measured in the dark shows the built-in potential contrast likely originating from the local material composition and/or dopant distribution (see Figure 3b,g). In the LT sample, the contact potential difference at grain boundaries is smaller than that at grain interiors in the dark condition. The contact potential contrast between grain boundaries and grain interiors becomes stronger when increasing the intensity of light illumination (see Figure 3b–e). Accordingly, it is apparent that grain boundaries from the LT sample have lower potential values than those at grain interiors as shown in Figure 2b. In contrast, contact potential difference values at grain boundaries are initially higher than those at grain interiors in the dark condition for the HT sample as shown in Figure 3g and remain higher with the illumination. To better illustrate the correlation between photovoltage and grain morphology, we overlaid the topography images with the photovoltage images (see Figure 2c,f). The high photovoltage profiles at grain boundaries of the HT sample are clearly observed in Figures 2 and 3. It is possible that the concentration of Cl at grain boundaries increases due to a smaller portion of grain boundary area in the HT sample assuming that the same amount of cadmium chloride (CdCl_2_) is injected into both the LT and HT CdTe samples during the Cl activation treatment. The higher concentration of Cl or higher processing temperature results in a more effective passivation of structural defects at grain boundaries in the HT sample. In the HT sample, we obtained a higher open-circuit voltage than that in the LT sample as shown in Table 1 and also observe the enhanced photovoltage at grain boundaries from the nanoscale measurement (i.e., Δ*V*_CPD_ in Figure 2). However, the reduced photocurrent in the HT sample counteracts further improvement of cell efficiency as shown in Table 1. This fact illustrates that CdTe samples should be optimized so that a grain size and a sample thickness are in certain ranges to enhance both photovoltage and photocurrent, by adjusting a sample preparation temperature and a Cl activation treatment condition, like the optimized CdTe cell in Table 1.

**Figure 4:**
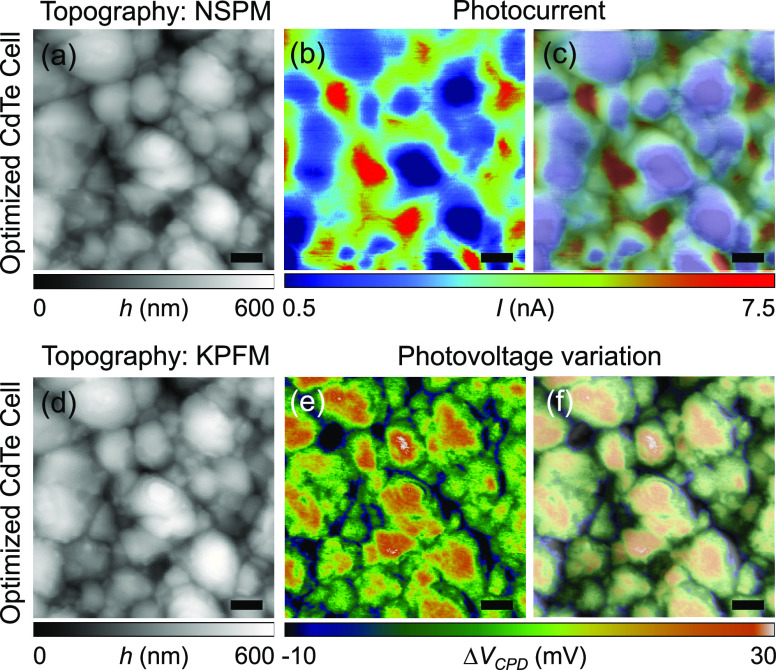
AFM topography images of the optimized CdTe sample obtained by (a) NSPM and (d) KPFM measurements. Both measurements were performed on the same location of the sample. (b) Measured photocurrent image. (c) AFM topography image-overlaid photocurrent image. (e) Measured photovoltage image. (f) AFM topography image-overlaid photovoltage image. Scale bar: 1 μm.

As already discussed, the macroscopic photovoltaic properties of the optimized CdTe solar cell in Table 1 are the best among all three CdTe cells. To examine this enhancement on nanoscale, we performed NSPM and KPFM measurements to obtain both local photovoltage and photocurrent (see Figure 4). These two measurements were performed at the same area of the sample, so we can more systemically evaluate nanoscopic photoresponses of polycrystalline CdTe solar cells based on multiple data sets. The optimized CdTe solar cell was first examined by NSPM. For NSPM measurements, we illuminated the sample surface within a near-field using a laser light injected through a tapered optical fiber having a small aperture (≈200 nm). The wavelength of the laser light is ≈520 nm. The end of the optical fiber is coated with metal, and the fiber is mounted on a quartz tuning fork-based AFM probe. The NSPM photocurrent image shows higher photocurrent at grain boundaries, meaning that the grain boundaries are the major carrier collection paths (see Figure 4c). We attribute the enhanced carrier collections at grain boundaries to the modified band structures and the resulting built-in electric field. The Fermi energy level of grain boundaries is higher than that of grain interiors. This can be explained by the fact that a p–n–p junction is formed around grain boundaries, as CdTe is a p-type semiconductor (i.e., p-type grain interiors and n-type grain boundaries). Thus, there exists a built-in electric field between grain boundaries and grain interiors, facilitating photogenerated minority carrier collection through grain boundaries.

We also obtained the KPFM photovoltage image from the same location of the sample (see Figure 4f). Contrary to the NSPM photocurrent image, lower photovoltage is observed near grain boundaries. It indicates that the loss of photogenerated carriers around grain boundaries is higher because of dangling bonds and other recombination sites at the grain boundaries. In addition, a relatively high Fermi energy level at grain boundaries confirms the different composition and doping leading to an enhanced recombination and reduced photovoltage compared with that in grain interiors, as shown in Figure 4f.^42^ As compared to the HT sample, the optimized sample exhibits a higher power conversion efficiency (see Table 1) in spite of the lower photovoltage at grain boundaries (see Figure 4f). The higher power conversion efficiency in the optimized CdTe cell is mainly caused by the highest photocurrent as shown in Table 1. Despite degraded photogenerated voltage,^54^Figure 4 illustrates that the grain boundaries can contribute to increasing photogenerated current by more effectively collecting carriers when CdTe solar cells are exposed to Cl and annealed at an appropriate temperature.^41,49,55,56^ It is apparent that the grain boundaries lead to two counteracting contributions: a built-in electric field helps to collect more photogenerated carriers, while the enhanced recombination impairs the photovoltage, as can be confirmed with Figure 4c,f. Thus, we need to combine all information we obtain (i.e., photocurrent and photovoltage maps obtained by NSPM and KPFM) to more accurately evaluate the role of CdTe grain boundaries and their impact on the overall device performance such as the power conversion efficiency.

**Figure 5:**
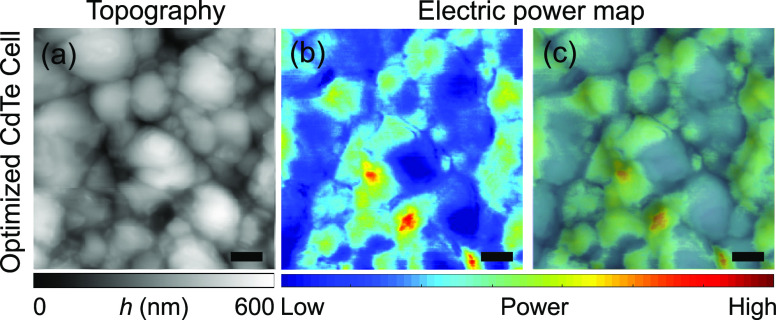
(a) Topography of the optimized CdTe sample. (b) Electric power map was created by multiplying photovoltage and photocurrent maps on the same location. (c) AFM topography image-overlaid electric power map. Scale bar: 1 μm.

In this regard, we created a nanoscale electric power map by correlating the photocurrent and photovoltage images shown in Figure 4 (see Figure 5). The power map does not quantitatively yield the spatially resolved nanoscale power conversion efficiency. First, it was obtained by multiplying the photocurrent and photovoltage values without considering the fill factor. Other limitations of such approach are discussed later in the manuscript. However, the product of photocurrent and photovoltage is still useful to holistically analyze the nanoscopic power generation and loss patterns in polycrystalline grain medium. While some grain boundaries still contribute to enhancing the electric power generation, we commonly observe that a higher electric power is generated within some grain interiors. Up to date, the beneficial role of CdTe grain boundaries in carrier collection has been suggested by many publications, mostly derived from a measurement of a single electrical quantity (e.g., photocurrent) and/or analytical calculations.^34,49,54^ As we consider both photocurrent and photovoltage for a more complete evaluation of the role of grain boundaries, we can specify the limits of the beneficial role of grain boundaries: the boosted electric power is observed at grain boundaries only if the carrier collection efficiency overcomes the recombination rate. Observing two independent electrical properties at the same location consequently yields a better insight into how solar cell microscopic grain structures affect macroscopic device characteristics. In addition, this helps to establish a precise macroscale photoresponse model of polycrystalline solar cells consisting of micro/nanoscale grain structures.

**Figure 6:**
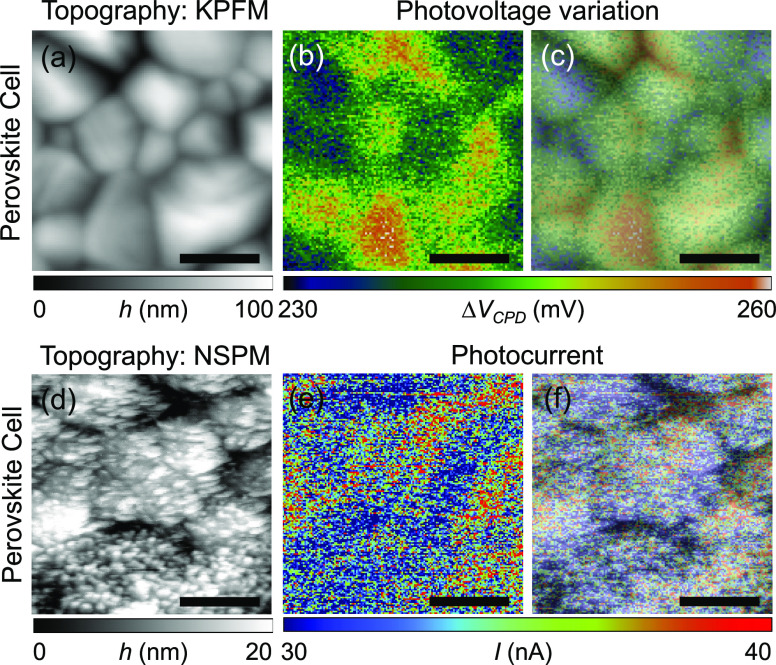
(a) AFM topography image of the perovskite solar cell obtained by KPFM. (b) Photovoltage image and (c) AFM topography image-overlaid photovoltage image. (d) AFM topography image of the perovskite solar cell obtained by NSPM. (e) Photocurrent image and (f) AFM topography image-overlaid photocurrent image. Scale bar: 500 nm.

Both KPFM and NSPM measurements can be readily used for characterization of solar cells made from different materials, including organic–inorganic hybrid perovskite solar cells. To prove this, the same measurement techniques were applied to examine the local photoresponse of a hybrid perovskite solar cell. A 400 nm thick methylammonium lead iodide (CH_3_NH_3_PbI_3_) perovskite layer is sandwiched between a 15 nm nickel oxide (NiO*_x_*) hole transport layer and an electron transport layer composed of a 50 nm thick zinc oxide nanoparticle (ZnO NP) layer and a 50 nm thick phenyl-C_61_-butyric acid methyl ester ([60]PCBM) film. A 120 nm Ag layer and a 280 nm indium tin oxide (ITO) layer form contacts to the electron transport layer and the hole transport layer, respectively. The whole cell structure was fabricated atop a soda-lime glass substrate. While some grain boundaries show enhanced photovoltage, we also measured high photovoltage at some other grain interiors as shown in Figure 6c. In addition, grain-to-grain photovoltage variations were observed. Based on the photovoltage measurement, it is not clear whether grain boundaries or grain interiors are dominating minority carrier recombination centers. The complex photovoltage trend observed in the perovskite solar cell can be attributed to the complex interplay between grain boundaries and grain interiors in this sample. This complexity arises from variations in defect densities, carrier recombination rates, and carrier transport properties across the perovskite film, as well as from the presence of different phases (e.g., PbI_2_), local compositional variations, and different degrees of passivation at grain boundaries and grain interiors.

Unlike the varying photovoltage distribution patterns, it is found that photocurrent is consistently enhanced near grain boundaries (see Figure 6f). This observation is consistent with some previous studies performed on perovskite solar cells.^35,39^ A certain amount of segregated lead iodide (PbI_2_) layers effectively acts as a barrier for holes.^39^ The barrier prevents holes from facing shallow carrier traps located near grain boundaries that are not passivated, ultimately reducing carrier recombination. Thus, the moderately segregated PbI_2_ layer near grain boundaries is the driving force to transport more photogenerated minority carriers at the grain boundaries.^57−60^ Furthermore, lead (Pb) in grain interiors and PbI_2_ in grain boundaries share the same coordination, so the existence of PbI_2_ near grain boundaries helps reduce the concentration of dangling bonds at the grain boundaries.^39^ It is worth mentioning that photogenerated carrier collections are significantly suppressed along grain boundaries if an excessive amount of PbI_2_ is segregated and stacked at grain boundaries (see Figure S2 in the Supporting Information for cross-sectional scanning electron microscopy (SEM) images).

In contrast to inorganic CdTe solar cells, the organic–inorganic hybrid perovskite solar cell we used for our study showed some degree of performance degradation under continuous light illumination. However, no significant compositional changes were found until ≈400 min of continuous light illumination under the AM1.5G spectrum.^39^ As our nanoscale scans were accomplished in a relatively very short period of time, the dynamics of PbI_2_ (e.g., PbI_2_ migration) was very limited in time and space. Furthermore, it is important to note that both KPFM and NSPM measurements are basically nondestructive measurement techniques. Nonetheless, to minimize any effects from light-induced degradation or PbI_2_ redistribution under light illumination for our KPFM and NSPM measurements and to fairly quantify and compare two different nanoscale electrical properties (i.e., photovoltage and photocurrent), KPFM and NSPM measurements were performed on two different areas where no prior light-induced measurements were carried out. This approach helps us to ensure that the PbI_2_ content remains relatively consistent during all measurements, allowing for a more accurate evaluation of nanoscale optoelectronic responses of a perovskite solar cell.

The heterogeneity in photovoltage across the scanned area of the organic–inorganic hybrid perovskite cell can be attributed to variation in material properties such as grain structures, defect distributions, and others. Perovskite materials exhibit a high degree of structural complexity and compositional variability, and therefore, the grain structure of perovskite solar cells can be significantly inconsistent. For example, the presence of segregated PbI_2_ near grain boundaries in the perovskite solar cell can have a considerable impact on photovoltage distribution. As already discussed, the existence of PbI_2_ near grain boundaries can effectively reduce photogenerated carrier recombination and alter the photovoltage distribution across the scanned area.

While we have demonstrated the capabilities of combining and correlating two AFM-based methods, KPFM and NSPM, the limitations of the measurement techniques should also be discussed. Surface contact potential information obtained by KPFM measurements does not necessarily represent the bulk optoelectronic properties and may be limited to the response of the surface region of samples. At worst, the contact potential would merely characterize the surface recombination, if unavoidable surface defect states are extremely high. Useful information regarding the local carrier collection and loss within the cells can be hidden in the optoelectronic response that represents the surface defect states. Thus, a further work to separate meaningful contact potential values from the surface recombination information is required. In addition, as the KPFM and NSPM measurement techniques use different light sources and methods, the obtained photoresponse may not be directly linked to quantify the nanoscale power conversion efficiency. While the NSPM technique is based on a local area light injection (i.e., near-field laser light injection through a tapered optical fiber having a diameter of several hundred nanometers) and a large area electrical property detection mechanism (i.e., current is collected through the top and bottom macroscale electrodes of devices), the KPFM technique injects light onto a relatively large area of the sample surface (i.e., far-field laser light illumination with a spot size of several millimeters) and detects electrical properties at a local area using a conductive cantilever. We note that the current–voltage measurements accomplished either by using a local contact of the order of the grain size or by photoconductive AFM cannot reliably represent the local power efficiency. In such measurements, the local contact collects photoexcited carriers generated over the area that is larger or much larger than the contact size depending on the photovoltaic technology and in-plane transport properties of photoexcited carriers. Thus, the approach described in the present paper represents the best approximation of the local efficiency in spite of the limitations discussed above.

Despite such limitations, this work shows the capability to comprehensively evaluate the role of grain boundaries in polycrystalline thin-film solar cells by correlating multiple nanoscale electrical properties. With further advancing nanoscale measurement techniques, developing multiple measurement techniques relying on similar carrier generation/collection mechanisms, and performing proper correlations, the multiple data sets can be used to accurately evaluate how the optical, electrical, and material factors affect nanoscopic electrical power generation and loss. This information is useful in determining the macroscopic behavior of solar cells, leading to a more in-depth interpretation of device operations, and eventually contributing to building an accurate macroscale operational model of polycrystalline solar cells.

## Conclusions

We investigated nanoscale optoelectronic responses of CdTe and perovskite solar cells using KPFM and NSPM measurements. We observed that grain boundaries of CdTe solar cells can simultaneously act both as carrier recombination centers and efficient carrier collectors. Grain boundaries are photogenerated minority carrier collectors due to the electric field, explaining the increased photocurrent near grain boundaries. However, they simultaneously also work as recombination centers due to the inherent surface detects and dangling bonds between the grains. Thus, we provided a map of nanoscale power, which incorporates both the photocurrent and photovoltage to evaluate how grain boundaries affect the power efficiency of the cells. We also found the correlations between the nanoscale and the macroscale CdTe photovoltaic properties based upon our measurements. It is found that the nanoscale information can support macroscale data sets, providing a path for an accurate macroscale polycrystalline solar cell operational model based on nanoscale information. For a perovskite solar cell, the beneficial role of grain interiors and grain boundaries based on the nanoscale photovoltage image is equivocal. However, based on the nanoscale photocurrent image, it is clear that the grain boundaries with a moderate amount of PbI_2_ efficiently collect photogenerated minority carriers.

## Supporting Information Available

Details regarding Cl segregation at grain boundaries in CdTe solar cells and distribution of PbI_2_ crystallites in perovskite solar cells (PDF)
