# Supplementary material for: Nanoscale Characterization of Photocurrent and Photovoltage in Polycrystalline Solar Cells
Source: J Phys Chem C Nanomater Interfaces. 2023 Jun 7;127(24):11429–37. doi: 10.1021/acs.jpcc.3c00239 (PMC10291557; doi:10.1021/acs.jpcc.3c00239)
Supplement: Supplementary file 1 — jp3c00239_si_001.pdf [file jp3c00239_si_001.pdf]

Supporting Information

Nanoscale Characterization of Photocurrent and Photovoltage in Polycrystalline Solar Cells

Dongheon Ha,[1,2,3,#,*] Yohan Yoon,[2,3,4,#,*] Ik Jae Park,[5] Luis Torres Cantu,[1] Aries Martinez,[1] and Nikolai Zhitenev[2]

1. Department of Physics, Eastern Illinois University, Charleston, IL 61920, USA
2. Physical Measurement Laboratory, National Institute of Standards and Technology, Gaithersburg, MD 20899, USA
3. Institute for Research in Electronics and Applied Physics, University of Maryland, College Park, MD 20742, USA
4. Department of Materials Science and Engineering, Korea Aerospace University, Goyang-si, Gyeonggi-do, 10540, Korea
5. Department of Materials Physics, Sookmyung Women's University, Seoul, 04310, Korea

# These authors equally contributed to this work.
* Corresponding authors: Dr. Dongheon Ha: dha@eiu.edu, Dr. Yohan Yoon: yyoon@kau.ac.kr

Energy dispersive X-ray spectroscopy in scanning transmission electron microscopy (EDX/STEM) line profiles of Cl in both HT and LT samples

EDX/STEM line profiles in Figure S1 confirm the segregation of Cl at grain boundaries in the HT CdTe cell. The Cl segregation at grain boundaries was not observed in the other samples.

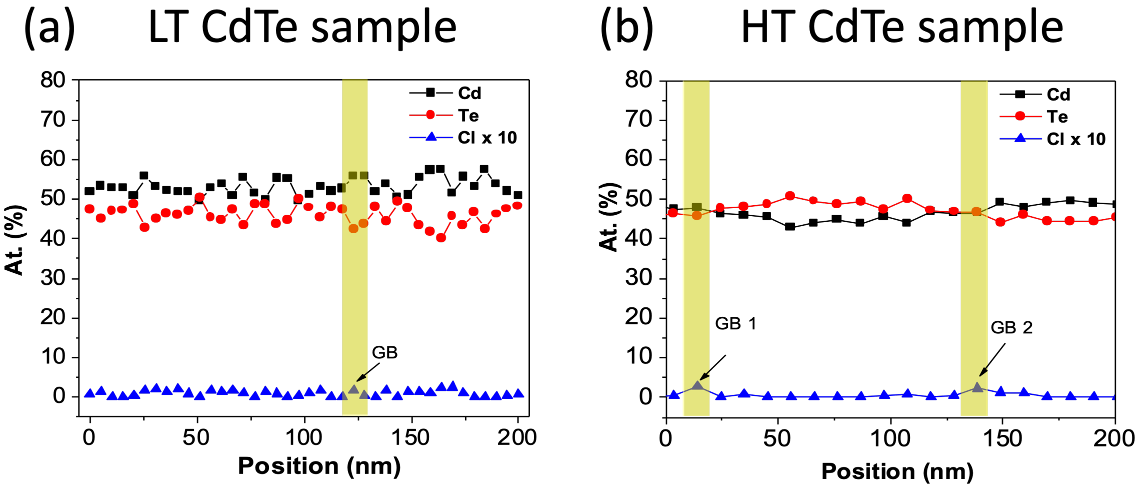

Figure S1. EDX/STEM line profile at grain boundaries of (a) a low temperature (LT) and (b) a high temperature (HT) CdTe samples. Segregated Cl is consistently detected only near grain boundaries of the HT CdTe sample.

Cross-sectional scanning electron microscopy (SEM) images of perovskite solar cells

As shown in Figure S2, the distribution of PbI_2 crystallites across the active region is quite different in the two samples prepared at two different temperatures (100 °C and 130 °C). PbI_2 is relatively uniformly distributed in the sample annealed at 100 °C, while it is frequently segregated and stacked along some grain boundaries in the sample annealed at 130 °C. The sample annealed at 100 °C in which there is a more uniform distribution of PbI_2 is used in our work to more purely quantify the role of micro-/nanoscopic grain structures on optoelectronic responses of a hybrid perovskite solar cell.

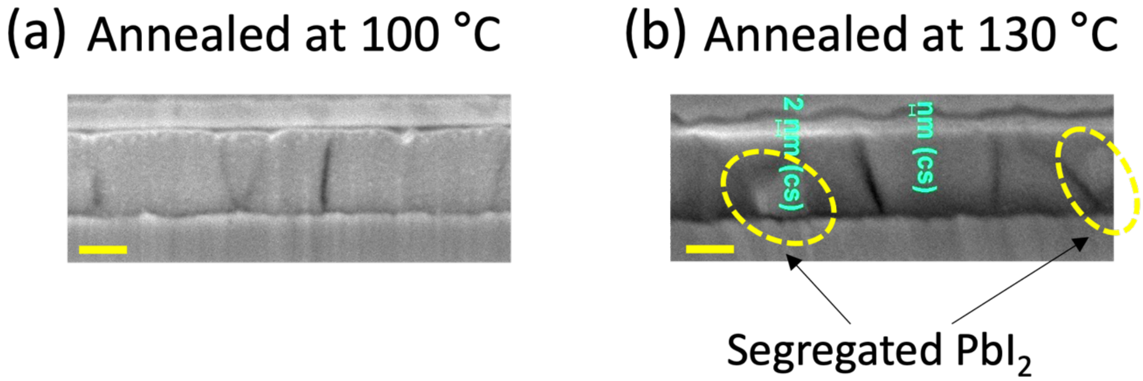

Figure S2. Cross-sectional SEM images of (a) a perovskite solar cell annealed at 100 °C and (b) a perovskite solar cell annealed at 130 °C. It is apparent that PbI_2 (bright spots in (b) indicated with yellow-dotted ovals) is excessively segregated at grain boundaries of the sample annealed at a higher temperature. In the sample annealed at 100 °C, the distribution of PbI_2 is very uniform across the active region of the sample. The green characters in (b) were generated by the SEM system while taking length measurements on the obtained image. Scale bars are 250 nm.
